# Supplementary material for: Amino Acid Profile Alterations in the Mother–Fetus System in Gestational Diabetes Mellitus and Macrosomia
Source: Int J Mol Sci. 2025 Aug 28;26(17):8351. doi: 10.3390/ijms26178351 (PMC12429083; doi:10.3390/ijms26178351)
Supplement: Supplementary file 1 [file ijms-26-08351-s001.zip › Supplementary tables.pdf]

Table S1. Levels of maternal venous serum aminoacids (nmol/mL) in each group and p-value, calculated by Kruskall-Wallis test. For p-value <0.05 according Kruskall-Wallis test p-value according Dunn test were calculated. P_1 – comparison control group and GDM +macrosomia group, p_2 – comparison control group and GDM +normosomia group, p_3 – comparison GDM + macrosomia and GDM +normosomia groups.

Aminoacids	Control	GDM +macrosomia	GDM+ normosomia	P	Pairwise comparison (p<0.05)
1-Methylhistidine	0 (0; 0)	0 (0; 0)	0 (0; 0)	0.63	-
2-Aminobutyric acid	3.2 (0; 12)	0 (0; 3.9)	1.6 (0; 7.2)	0.47	-
3-Methylhistidine	0 (0; 0)	0 (0; 0)	0 (0; 0)	0.43	-
4-Aminobutyric acid	0.57 (0.23; 1.1)	0.37 (0; 0.6)	0.38 (0.13; 0.91)	0.10	-
5-Hydroxylysine	0.35 (0; 1.8)	2.2 (0; 3.5)	2.6 (0.41; 5.6)	0.13	-
Alanine	2293 (931; 4560)	0 (0; 2296)	965 (203; 2792)	0.08	-
Arginine	0 (0; 0)	0 (0; 0)	0 (0; 0)	0.32	-
Asparagine	71 (46; 128)	26 (18; 73)	57 (33; 86)	0.03	$p_1 = 0.01$ $p_2 = 0.22$ $p_3 = 0.05$
Aspartic acid	23 (2.5; 44)	6.9 (0; 25)	15 (5.5; 37)	0.37	-
β-Alanine	0.23 (0; 0.68)	0 (0; 0.12)	0 (0; 0.56)	0.23	-
Citrulline	0 (0; 0)	0 (0; 0)	0 (0; 0)	0.75	-
Glutamic acid	0 (0; 0)	0 (0; 0)	0 (0; 0)	0.97	-
Glutamine	18246 (7379; 22725)	9545 (4606; 15071)	11376 (8877; 16145)	0.09	-
Glycine	0 (0; 1035)	0 (0; 0)	0 (0; 2365)	0.30	-
Histidine	1104 (321; 1664)	621 (0; 1027)	265 (32; 946)	0.09	-
Homocitrulline	0.74 (0; 9)	5.1 (0; 9.4)	1.4 (0; 4.2)	0.36	-
Isoleucine	0.24 (0; 51)	0 (0; 49)	5 (0; 105)	0.77	-
Leucine	0 (0; 173)	0 (0; 7.3)	0 (0; 260)	0.47	-
Lysine	729 (302; 1466)	305 (0; 477)	648 (117; 1060)	0.06	-
Methionine	0 (0; 8)	0 (0; 0)	0 (0; 9.2)	0.30	-
Ornithine	0 (0; 0)	0 (0; 0)	0 (0; 67)	0.62	-
Phenylalanine	78 (0; 216)	0 (0; 94)	0 (0; 181)	0.12	-
Proline	0 (0; 0)	0 (0; 0)	0 (0; 0)	0.39	-
Sarcosine	0 (0; 12)	0 (0; 41)	0 (0; 4.2)	0.64	-
Serine	0 (0; 0)	0 (0; 0)	0 (0; 248)	0.05	-
Threonine	1392 (687; 2109)	1158 (489; 2032)	1033 (661; 2249)	0.88	-
Tryptophan	0 (0; 21)	0 (0; 0)	0 (0; 0)	0.09	-
Tyrosine	0 (0; 0)	0 (0; 0)	0 (0; 0)	0.45	-
Valine	0 (0; 0)	0 (0; 0)	0 (0; 0)	0.85	-

Table S2. Levels of umbilical cord blood aminoacids (nmol/mL) in each group and p-value, calculated by Kruskall-Wallis test. For p-value <0.05 according Kruskall-Wallis test p-value according Dunn test were calculated. P_1 – comparison control group and GDM +macrosomia group, p_2 – comparison control group and GDM +normosomia group, p_3 – comparison GDM + macrosomia and GDM +normosomia groups.

Aminoacids	Control	GDM +macrosomia	GDM+ normosomia	P	Pairwise comparison (p<0.05)
1-Methylhistidine	3.9 (3.2; 4.2)	3.7 (3.2; 4.7)	3.6 (3.3; 4)	0.74	-
3-Methylhistidine	0.31 (0.2; 0.59)	0.32 (0.19; 0.50)	0.36 (0.18; 0.66)	0.85	-
Alanine	262 (221; 356)	304 (171; 348)	317 (222; 416)	0.75	-
2-Aminobutyric acid	16 (13; 19)	18 (16; 21)	19 (13; 21)	0.20	-
Arginine	31 (21; 39)	27 (20; 40)	31 (27; 42)	0.46	-
Asparagine	43 (36; 52)	44 (33; 55)	40 (34; 46)	0.44	-
Aspartic acid	19 (16; 29)	18 (15; 32)	18 (16; 26)	0.78	-
β-Alanine	5.4 (4.4; 6.3)	5.1 (4.4; 5.8)	5.2 (4.6; 5.6)	0.68	-
Citrulline	6.8 (5.7; 7.7)	6.3 (5.2; 7.2)	5.5 (5; 6.9)	0.21	-
Glutamic acid	144 (128; 191)	158 (120; 225)	141 (131; 172)	0.77	-
Glutamine	201 (169; 238)	173 (151; 252)	178 (169; 202)	0.55	-
Glycine	331 (292; 372)	322 (289; 340)	331 (308; 390)	0.58	-
Histidine	151 (134; 165)	144 (133; 159)	143 (122; 161)	0.43	-
Isoleucine	39 (36; 47)	49 (42; 54)	46 (39; 53)	0.03	p_1 = 0.02<br>p_2= 0.12<br>p_3 = 0.71
Leucine	137 (129; 156)	152 (127; 171)	150 (129; 170)	0.42	-
Lysine	533 (499; 581)	487 (452; 534)	533 (494; 584)	0.13	-
Methionine	70 (61; 77)	65 (53; 75)	60 (56; 70)	0.13	-
Ornithine	205 (180; 222)	186 (161; 225)	195 (172; 220)	0.55	-
Phenylalanine	89 (87; 101)	85 (81; 92)	87 (80; 96)	0.37	-
Proline	235 (208; 262)	221 (200; 237)	221 (201; 226)	0.27	-
Serine	13 (11; 15)	15 (12; 17)	13 (12; 15)	0.28	-
Threonine	244 (217; 295)	308 (241; 332)	258 (199; 278)	0.10	-
Tryptophan	97 (89; 103)	93 (86; 106)	106 (86; 116)	0.44	-
Tyrosine	38 (36; 43)	38 (31; 46)	37 (34; 46)	0.98	-
Valine	197 (177; 218)	208 (193; 224)	210 (194; 245)	0.32	-

Table S3. Levels of amniotic fluid aminoacids (nmol/mL) in each group and p-value, calculated by Kruskall-Wallis test. For p-value <0.05 according Kruskall-Wallis test p-value according Dunn test were calculated. P_1 – comparison control group and GDM +macrosomia group, p_2 – comparison control group and GDM +normosomia group, p_3 – comparison GDM + macrosomia and GDM +normosomia groups.

Aminoacids	Control	GDM +macrosomia	GDM+ normosomia	P	Pairwise comparison (p<0.05)
1-Methylhistidine	0.08 (0.069; 0.12)	0.089 (0.077; 0.11)	0.094 (0.075; 0.12)	0.69	-
3-Methylhistidine	0.087 (0.039; 0.14)	0.11 (0.057; 0.15)	0.15 (0.098; 0.22)	0.17	-
5-Hydroxylysine	0.017 (0.014; 0.02)	0.02 (0.018; 0.024)	0.022 (0.014; 0.027)	0.28	-
Alanine	0.59 (0.46; 0.69)	0.7 (0.5; 0.95)	0.68 (0.57; 1.1)	0.13	-
2-Aminobutyric acid	0.031 (0.017; 0.047)	0.039 (0.023; 0.052)	0.032 (0.014; 0.054)	0.59	-
Arginine	0.23 (0.19; 0.25)	0.22 (0.2; 0.35)	0.28 (0.24; 0.45)	0.02	p_1 = 0.40<br>p_2= 0.006

					p_3 = 0.20
Asparagine	0 (0; 0.0028)	0 (0; 0.014)	0 (0; 0.021)	0.63	-
Aspartic acid	0.041 (0.029; 0.051)	0.055 (0.031; 0.13)	0.075 (0.05; 0.14)	0.02	p_1 = 0.13<br>p_2= 0.008<br>p_3 = 0.57
β-Alanine	0.031 (0.021; 0.049)	0.035 (0.03; 0.051)	0.041 (0.028; 0.058)	0.37	-
Citrulline	0.087 (0.075; 0.1)	0.13 (0.068; 0.19)	0.11 (0.077; 0.21)	0.20	-
4-Aminobutyric acid	0.027 (0.021; 0.034)	0.026 (0.022; 0.039)	0.039 (0.027; 0.053)	0.04	p_1 = 1.00<br>p_2= 0.02<br>p_3 = 0.11
Glutamic acid	1.7 (1.2; 2.1)	1.9 (1.2; 2.6)	2.6 (1.9; 3.1)	0.03	p_1 = 0.50<br>p_2= 0.02<br>p_3 = 0.11
Glutamine	0.092 (0.066; 0.1)	0.094 (0.079; 0.2)	0.15 (0.083; 0.24)	0.03	p_1 = 0.17<br>p_2= 0.02<br>p_3 = 0.71
Glycine	0.18 (0.13; 0.22)	0.19 (0.16; 0.27)	0.23 (0.15; 0.33)	0.18	-
Histidine	1.4 (1; 1.6)	1.4 (1; 2.1)	1.5 (1.1; 2.2)	0.24	-
Isoleucine	0.056 (0.041; 0.073)	0.077 (0.044; 0.16)	0.088 (0.06; 0.2)	0.01	p_1 = 0.21<br>p_2= 0.006<br>p_3 = 0.37
Leucine	0.63 (0.51; 0.74)	0.91 (0.54; 1.9)	1.1 (0.71; 2.8)	0.01	p_1 = 0.21<br>p_2= 0.007<br>p_3 = 0.38
Lysine	0.89 (0.72; 1.3)	1 (0.71; 1.3)	1.1 (0.89; 1.4)	0.27	-
Methionine	0.073 (0.059; 0.085)	0.079 (0.059; 0.15)	0.1 (0.067; 0.15)	0.16	-
Ornithine	0.27 (0.23; 0.34)	0.29 (0.22; 0.66)	0.42 (0.32; 0.79)	0.02	p_1 = 0.42<br>p_2= 0.007<br>p_3 = 0.22
Phenylalanine	0.24 (0.18; 0.31)	0.25 (0.2; 0.52)	0.39 (0.25; 0.52)	0.09	-
Proline	2.1 (1.4; 3.9)	2.6 (1.8; 3.7)	3.1 (2; 4)	0.20	-
Sarcosine	0.018 (0.014; 0.022)	0.019 (0.013; 0.024)	0.019 (0.014; 0.025)	0.79	-
Serine	0.032 (0.02; 0.04)	0.043 (0.033; 0.098)	0.056 (0.023; 0.087)	0.047	p_1 = 0.06<br>p_2= 0.07<br>p_3 = 1.00
Threonine	0.014 (0.013; 0.025)	0.021 (0.016; 0.025)	0.021 (0.016; 0.028)	0.07	-
Tryptophan	0 (0; 0.027)	0.012 (0; 0.033)	0.028 (0; 0.036)	0.28	-
Tyrosine	0.25 (0.21; 0.32)	0.27 (0.21; 0.45)	0.33 (0.24; 0.54)	0.12	-
Valine	0.26 (0.21; 0.31)	0.28 (0.23; 0.47)	0.32 (0.25; 0.65)	0.07	-

Table S5. Clinical parameters in each group. P-value calculated by Kruskall-Wallis test

**Clinical parameter**	**Group**		**Value**	**P**
Age, years	GDM «+» (n=53)	Macrosomia «+» (n=23)	33.5 (29; 37)	p=0.64
		Macrosomia «-» (n=30)	33.5 (30; 37)	

	GDM «-» (n=41)	Macrosomia «-» (n=41)	34 (27; 35)	
Weight before pregnancy, kg	GDM «+» (n=53)	Macrosomia «+» (n=23)	84 (66; 96)	**p=0.006***
		Macrosomia «-» (n=30)	63 (55; 67)	
	GDM «-» (n=41)	Macrosomia «-» (n=41)	59 (54; 63)	
Height, cm	GDM «+» (n=53)	Macrosomia «+» (n=23)	170 (164; 180)	p= 0.33
		Macrosomia «-» (n=30)	168 (164; 170)	
	GDM «-» (n=41)	Macrosomia «-» (n=41)	167 (164; 171)	
BMI before pregnancy, kg/m2	GDM «+» (n=53)	Macrosomia «+» (n=23)	29 (25; 35)	**p< 0.001***
		Macrosomia «-» (n=30)	28 (24; 32)	
	GDM «-» (n=41)	Macrosomia «-» (n=41)	22 (21; 24)	
Weight gain at birth, kg	GDM «+» (n=53)	Macrosomia «+» (n=23)	14 (11; 16)	**p= 0.002***
		Macrosomia «-» (n=30)	11 (9; 14)	
	GDM «-» (n=41)	Macrosomia «-» (n=41)	13 (11; 16)	
Nulliparous women, n (%)	GDM «+» (n=53)	Macrosomia «+» (n=23)	7 (30%)	p= 0.90
		Macrosomia «-» (n=30)	13 (43%)	
	GDM «-» (n=41)	Macrosomia «-» (n=41)	16 (39%)	

Duration of delivery, weeks	GDM «+» (n=53)	Macrosomia «+» (n=23)	39 (38; 39)	p= 0.33
		Macrosomia «-» (n=30)	39 (38; 39)	
	GDM «-» (n=41)	Macrosomia «-» (n=41)	39 (38; 39)	
Operative delivery, number of patients (%)	GDM «+» (n=53)	Macrosomia «+» (n=23)	16 (70%)	**p= 0.003***
		Macrosomia «-» (n=30)	17 (56%)	
	GDM «-» (n=41)	Macrosomia «-» (n=41)	10 (24%)	
Gender of the newborn (number of boys)	GDM «+» (n=53)	Macrosomia «+» (n=23)	12 (52%)	p=0.49
		Macrosomia «-» (n=30)	20 (67%)	
	GDM «-» (n=41)	Macrosomia «-» (n=41)	19 (54%)	
Weight of newborn, g	GDM «+» (n=53)	Macrosomia «+» (n=23)	4110 (3905; 4165)	**p< 0.001***
		Macrosomia «-» (n=30)	3245 (3025; 3507)	
	GDM «-» (n=41)	Macrosomia «-» (n=41)	3380 (3135; 3560)	
Assessment of the newborn according to the Apgar scale - 1 minute, number of patients (%)	GDM «+» (n=53)	Macrosomia «+» (n=23)	8 (8; 8)	**p=0.008***
		Macrosomia «-» (n=30)	8 (8; 8)	
	GDM «-» (n=41)	Macrosomia «-» (n=41)	9 (8; 9)	

Assessment of the newborn according to the Apgar scale - 5 minutes, number of patients (%)	GDM «+» (n=53)	Macrosomia «+» (n=23)	9 (9; 9)	p=0.46
		Macrosomia «-» (n=30)	9 (9; 9)	
	GDM «-» (n=41)	Macrosomia «-» (n=41)	9 (9; 9)	
Placent width, mm	GDM «+» (n=53)	Macrosomia «+» (n=23)	40 (37; 40)	**p= 0.007***
		Macrosomia «-» (n=30)	37 (35; 38)	
	GDM «-» (n=41)	Macrosomia «-» (n=41)	38 (37; 40)	
Height of water pocket, mm	GDM «+» (n=53)	Macrosomia «+» (n=23)	70 (55; 85)	**p= 0.03***
		Macrosomia «-» (n=30)	56 (45; 73)	
	GDM «-» (n=41)	Macrosomia «-» (n=41)	50 (35; 65)	
HGB, g/L (Reference interval 110-139 g/L)	GDM «+» (n=53)	Macrosomia «+» (n=23)	118 (113; 127)	p=0.57
		Macrosomia «-» (n=30)	113.5 (109; 125)	
	GDM «-» (n=41)	Macrosomia «-» (n=41)	117 (109; 123)	
PLT, 10^{*9}/L (Reference interval 118-340 10^{*9}/L)	GDM «+» (n=53)	Macrosomia «+» (n=23)	248 (190; 268)	p=0.58
		Macrosomia «-» (n=30)	221 (196; 270)	
	GDM «-» (n=41)	Macrosomia «-» (n=41)	221 (185; 254)	
	GDM «+» (n=53)	Macrosomia «+» (n=23)	9.51 (8.18; 11.68)	

WBC, 10^{*9}/L (Reference interval 6,02-14,61 10^{*9}/L)		Macrosomia «-» (n=30)	9.04 (7.86; 10.18)	p=0.64
	GDM «-» (n=41)	Macrosomia «-» (n=41)	8.93 (8.32; 10.94)	
RBC, 10^{*12}/L (Reference interval 3,27-4,63 10^{*12}/L)	GDM «+» (n=53)	Macrosomia «+» (n=23)	4.09 (3.86; 4.3)	p=0.67
		Macrosomia «-» (n=30)	3.98 (3.79; 4.24)	
	GDM «-» (n=41)	Macrosomia «-» (n=41)	4.04 (3.71; 4.2)	
Glucose, mmol/L (Reference interval 3,9-5,0 mmol/L)	GDM «+» (n=53)	Macrosomia «+» (n=23)	4.4 (3.9; 4.7)	**p= 0.002***
		Macrosomia «-» (n=30)	4.3 (3.9; 4.9)	
	GDM «-» (n=41)	Macrosomia «-» (n=41)	3.9 (3.5; 4.1)	
ALT, unit/L (Reference interval 0-40 unit/L)	GDM «+» (n=53)	Macrosomia «+» (n=23)	11 (8.2; 17.4)	p=0.83
		Macrosomia «-» (n=30)	11.7 (8.5; 17.5)	
	GDM «-» (n=41)	Macrosomia «-» (n=41)	11.5 (9.2; 13.2)	
AST, unit/L (Reference interval 0-40 unit/L)	GDM «+» (n=53)	Macrosomia «+» (n=23)	20 (13.2; 24.6)	p=0.28
		Macrosomia «-» (n=30)	14.9 (12.9; 19.2)	
	GDM «-» (n=41)	Macrosomia «-» (n=41)	18 (14; 20.9)	
Total proteins, g/L	GDM «+» (n=53)	Macrosomia «+» (n=23)	62.7 (60.7; 67.9)	p=0.73
		Macrosomia «-» (n=30)	65.5 (62.1; 67.6)	

(Reference interval 55,8-71,2 g/L)	GDM «-» (n=41)	Macrosomia «-» (n=41)	64.2 (61.5; 67.4)	
Fibrinogen, g/L (Reference interval 4,8-6,0 g/L)	GDM «+» (n=53)	Macrosomia «+» (n=23)	4.4 (3.5; 4.9)	**p= 0.02***
		Macrosomia «-» (n=30)	65.5 (62.1; 67.6)	
	GDM «-» (n=41)	Macrosomia «-» (n=41)	64.2 (61.5; 67.4)	
INR (Reference interval 0,8-1,2)	GDM «+» (n=53)	Macrosomia «+» (n=23)	0.9 (0.8; 0.9)	p=0.47
		Macrosomia «-» (n=30)	0.9 (0.8; 0.9)	
	GDM «-» (n=41)	Macrosomia «-» (n=41)	0.9 (0.8; 0.9)	
APThT, s (Reference interval 25,0-38,0 s)	GDM «+» (n=53)	Macrosomia «+» (n=23)	27.2 (24.6; 28.2)	p=0.40
		Macrosomia «-» (n=30)	26.4 (25.3; 27.4)	
	GDM «-» (n=41)	Macrosomia «-» (n=41)	27.5 (25.2; 28.6)	

Table S6. Analyzed Amino Acids and Corresponding Internal Standards

Analyte	Internal Standard
1-Methylhistidine	Phenylalanine-d5
3-Methylhistidine	Phenylalanine-d5
5-Hydroxylysine	Ornithine-d2
Alanine	Ornithine-d2
2-Aminobutyric acid	Ornithine-d2
Arginine	Arginine-d3
Asparagine	Leucine-d3
Aspartic acid	Ornithine-d2
β-Alanine	Ornithine-d2

Citrulline	Citrulline-d2
4-Aminobutyric acid	Ornithine-d2
Glutamic acid	Ornithine-d2
Glutamine	Ornithine-d2
Glycine	Ornithine-d2
Histidine	Phenylalanine-d5
Homocitrulline	Citrulline-d2
Isoleucine	Leucine-d3
Leucine	Leucine-d3
Lysine	Arginine-d3
Methionine	Methionine-d3
Norvaline	Ornithine-d2
Ornithine	Ornithine-d2
Phenylalanine	Phenylalanine-d5
Proline	Phenylalanine-d5
Sarcosine	Ornithine-d2
Serine	Ornithine-d2
Threonine	Ornithine-d2
Tryptophan	Phenylalanine-d5
Tyrosine	Tyrosine-d4
Valine	Leucine-d3
