# Supplementary material for: Amino Acid Profile Alterations in the Mother–Fetus System in Gestational Diabetes Mellitus and Macrosomia
Source: Int J Mol Sci. 2025 Aug 28;26(17):8351. doi: 10.3390/ijms26178351 (PMC12429083; doi:10.3390/ijms26178351)
Supplement: Supplementary file 1 [file ijms-26-08351-s001.zip › Table S4.pdf]

Table S4: Alteration of metabolomic profile in system “mother-fetus”. M is maternal sefum venous blood, F is serum cord blood, AF is amniotic fluid. p without * - is comparing serum venous blood, p* - comparing serum cord blood, p** - comparing amniotic fluid.

Metabolite	Control			GDM			P-value	Normosomia			Macrosomia			P-value	Main changes in GDM	Association with macrosomia
	M, nmol/mL	F, nmol/mL	Af, rel. u.	M, nmol/mL	F, nmol/mL	AF, rel. u.		M, nmol/mL	F, nmol/mL	AF, rel. u.	M, nmol/mL	F, nmol/mL	AF, rel. u.			
5-OH-Lysine	↓ 0.35 (0; 1.8)	- 0 (0; 0)	- 0.017 (0.014; 0.02)	↑ 2.6 (0; 4.6)	- 0 (0; 0)	- 0.019 (0.015; 0.025)	p=0.05, p*=1, p**=0.2	- 1.3 (0; 3.9)	- 0 (0; 0)	- 0.017 (0.014; 0.024)	- 2.2 (0.49; 3.5)	- 0 (0; 0)	- 0.021 (0.018; 0.023)	p=0.49, p*=1, p**=0.13	Increase in serum and AF	Non-specific
Alanine	↑ 2293 (931; 4560)	- 262 (221; 356)	↓ 0.59 (0.46; 0.69)	↓ 843 (0; 2126)	- 304 (212; 358)	↑ 0.7 (0.53; 1.2)	p=0.01, p*=0.77, p**=0.02	- 1487 (0; 3936)	- 267 (220; 359)	- 0.63 (0.51; 0.99)	- 167 (0; 2296)	- 304 (186; 358)	- 0.71 (0.53; 0.98)	p=0.13, p*=0.86, p**=0.48	Decreased in serum, increased in AF	Non-specific
Arginine	- 0 (0; 0)	- 31 (21; 39)	↓ 0.23 (0.19; 0.25)	- 0 (0; 0)	- 29 (25; 40)	↑ 0.27 (0.21; 0.45)	p=0.82, p*=0.67, p**=0.007	- 0 (0; 0)	- 30 (22; 40)	- 0.24 (0.19; 0.33)	- 0 (0; 0)	- 27 (23; 40)	- 0.22 (0.2; 0.35)	p=0.30, p*=0.63, p**=1	Increase in AF	Increased in GDM without macrosomia
Asparagine	↑ 71 (46; 128)	- 43 (36; 52)	- 0 (0; 0.0028)	↓ 35 (22; 75)	- 41 (33; 49)	- 0 (0; 0.019)	p=0.01, p*=0.30, p**=0.16	↑ 60 (33; 92)	- 41 (36; 49)	- 0 (0; 0.014)	↓ 30 (21; 73)	- 41 (36; 51)	- 0 (0; 0.014)	p=0.04, p*=0.77, p**=0.79	Decreased in serum	Decreased in serum
Aspartic Acid	- 23 (2.5; 44)	- 19 (16; 29)	↓ 0.041 (0.029; 0.051)	- 13 (0; 43)	- 19 (16; 26)	↑ 0.067 (0.036; 0.16)	p=0.50, p*=0.61, p**=0.003	- 18 (3.3; 44)	- 19 (16; 28)	- 0.05 (0.031; 0.088)	- 13 (0; 35)	- 18 (15; 27)	- 0.055 (0.031; 0.13)	p=0.43, p*=0.70, p**=0.60	Increase in AF	Increase in AF in GDM + macrosomia
β-Alanine	↑ 0.23 (0; 0.68)	- 5.4 (4.4; 6.3)	- 0.031 (0.021; 0.049)	↓ 0 (0; 0.49)	- 5.2 (4.5; 5.8)	- 0.04 (0.028; 0.058)	p=0.14, p*=0.54, p**=0.14	- 0.044 (0; 0.62)	- 5.3 (4.5; 6.1)	- 0.037 (0.023; 0.055)	- 0 (0; 0.12)	- 5 (4.4; 5.5)	- 0.035 (0.028; 0.049)	p=0.08, p*=0.27, p**=0.96	Decreased in serum	Non-specific
Citrulline	- 0 (0; 0)	- 6.8 (5.7; 7.7)	↓ 0.087 (0.075; 0.1)	- 0 (0; 0)	- 6.2 (5; 6.9)	↑ 0.13 (0.073; 0.19)	p=0.38, p*=0.09, p**=0.046	- 0 (0; 0)	- 6.4 (5.3; 7.5)	- 0.093 (0.073; 0.15)	- 0 (0; 0)	- 6.3 (5.8; 7.4)	- 0.12 (0.072; 0.19)	p=0.96, p*=0.96, p**=0.41	Increase in AF	Non-specific
γ-Aminobutyric acid	↑ 0.57 (0.23; 1.1)	- 0 (0; 0)	- 0.027 (0.021; 0.034)	↓ 0.33 (0.054; 0.66)	- 0 (0; 0)	- 0.031 (0.024; 0.053)	p=0.045, p*=1, p**=0.07	- 0.44 (0.13; 1)	- 0 (0; 0)	- 0.03 (0.023; 0.042)	- 0.4 (0; 0.6)	- 0 (0; 0)	- 0.028 (0.022; 0.039)	p=0.14, p*=1, p**=0.82	Decreased in serum	Non-specific
Glutamic Acid	- 0 (0; 0)	- 144 (128; 191)	↓ 1.7 (1.2; 2.1)	- 0 (0; 0)	- 148 (131; 191)	↑ 2.4 (1.7; 3.8)	p=1, p*=0.65, p**=0.006	- 0 (0; 0)	- 144 (131; 189)	- 1.9 (1.4; 2.9)	- 0 (0; 0)	- 167 (129; 211)	- 2.1 (1.2; 3.1)	p=0.66, p*=0.47, p**=0.90	Increase in AF	Non-specific
Glutamine	↑ 18246 (7379; 22725)	- 201 (169; 238)	↓ 0.092 (0.066; 0.1)	↓ 10811 (6434; 16866)	- 178 (164; 217)	↑ 0.11 (0.077; 0.24)	p=0.04, p*=0.29, p**=0.01	- 13749 (7352; 20587)	- 187 (170; 225)	- 0.1 (0.068; 0.18)	- 10247 (4878; 16934)	- 190 (155; 258)	- 0.1 (0.083; 0.19)	p=0.28, p*=0.81, p**=0.37	Decreased in serum, increased in AF	Non-specific
Histidine	↑ 1104 (321; 1664)	- 151 (134; 165)	↓ 1.4 (1; 1.6)	↓ 220 (0; 962)	- 143 (129; 157)	↑ 1.6 (1.1; 2.2)	p=0.003, p*=0.09, p**=0.02	- 533 (0; 1364)	- 146 (131; 163)	- 1.5 (1; 1.9)	- 306 (0; 1027)	- 144 (132; 162)	- 1.4 (1.1; 2.1)	p=0.34, p*=0.81, p**=0.84	Decreased in serum, increased in AF	Non-specific
Homocitrulline	↓ 0.74 (0; 9)	- 0 (0; 0)	- 0 (0; 0)	↑ 3.2 (0; 6.6)	- 0 (0; 0)	- 0 (0; 0)	p=0.94, p*=1, p**=1	- 1.7 (0; 8.1)	- 0 (0; 0)	- 0 (0; 0)	- 2.9 (0; 7.6)	- 0 (0; 0)	- 0 (0; 0)	p=0.86, p*=1, p**=1	Increase in serum	Increased in serum in macrosomia + GDM
Isoleucine	- 0.24 (0; 51)	↓ 39 (36; 47)	↓ 0.056 (0.041; 0.073)	- 0 (0; 39)	↑ 45 (41; 53)	↑ 0.088 (0.052; 0.19)	p=0.7, p*=0.01, p**=0.004	- 0 (0; 50)	↓ 42 (38; 50)	- 0.061 (0.045; 0.1)	- 0 (0; 31)	↑ 45 (42; 54)	- 0.077 (0.045; 0.16)	p=0.53, p*=0.04, p**=0.52	Increase in umbilical cord blood and AF	Increase in cord blood
Leucine	- 0 (0; 173)	- 137 (129; 156)	↓ 0.63 (0.51; 0.74)	- 0 (0; 131)	- 150 (130; 170)	↑ 1 (0.6; 2.1)	p=0.49, p*=0.14, p**=0.005	- 0 (0; 160)	- 139 (130; 162)	- 0.69 (0.55; 1.7)	- 0 (0; 7.3)	- 152 (140; 169)	- 0.92 (0.54; 1.9)	p=0.23, p*=0.29, p**=0.59	Increase in AF	Non-specific
Lysine	↑ 729 (302; 1466)	- 533 (499; 581)	↓ 0.89 (0.72; 1.3)	↓ 324 (22; 735)	- 507 (462; 560)	↑ 1.1 (0.87; 1.5)	p=0.03, p*=0.17, p**=0.04	↑ 543 (45; 1352)	- 532 (480; 580)	- 1.1 (0.83; 1.4)	↓ 288 (0; 477)	- 489 (451; 540)	- 1.1 (0.71; 1.3)	p=0.03, p*=0.05, p**=0.67	Decreased in serum, increased in AF	Decreased in serum
Ornithine	- 0 (0; 0)	- 205 (180; 222)	↓ 0.27 (0.23; 0.34)	- 0 (0; 0)	- 192 (172; 221)	↑ 0.38 (0.24; 0.7)	p=0.91, p*=0.26, p**=0.01	- 0 (0; 4.6)	- 202 (176; 222)	- 0.31 (0.24; 0.54)	- 0 (0; 0)	- 194 (171; 226)	- 0.32 (0.22; 0.66)	p=0.31, p*=0.64, p**=0.78	Increase in AF	Increase in AF in GDM + normosomia
Phenylalanine	↑ 78 (0; 216)	- 89 (87; 101)	↓ 0.24 (0.18; 0.31)	↓ 0 (0; 124)	- 86 (81; 95)	↑ 0.35 (0.21; 0.6)	p=0.06, p*=0.22, p**=0.03	- 47 (0; 218)	- 89 (82; 99)	- 0.27 (0.19; 0.42)	- 0 (0; 109)	- 88 (81; 92)	- 0.26 (0.22; 0.52)	p=0.14, p*=0.56, p**=0.89	Decreased in serum, increased in AF	Non-specific
Proline	- 0 (0; 0)	- 235 (208; 262)	↓ 2.1 (1.4; 3.9)	- 0 (0; 0)	- 220 (195; 236)	↑ 3.3 (1.9; 4.7)	p=0.16, p*=0.093, p**=0.02	- 0 (0; 0)	- 222 (199; 248)	- 3.1 (1.8; 4.2)	- 0 (0; 0)	- 225 (203; 261)	- 3 (1.9; 4.3)	p=0.23, p*=0.89, p**=0.77	Increase in AF	Non-specific
Serine	- 0 (0; 0)	- 13 (11; 15)	↓ 0.032 (0.02; 0.04)	- 0 (0; 0)	- 13 (12; 15)	↑ 0.05 (0.022; 0.079)	p=0.75, p*=0.35, p**=0.02	- 0 (0; 29)	- 13 (11; 15)	- 0.035 (0.019; 0.058)	- 0 (0; 0)	- 15 (12; 16)	- 0.041 (0.031; 0.098)	p=0.40, p*=0.21, p**=0.13	Increase in AF	Increase in AF in GDM + macrosomia
Threonine	- 1392 (687; 2109)	- 244 (217; 295)	↓ 0.014 (0.013; 0.025)	- 1010 (491; 2059)	- 267 (215; 308)	↑ 0.022 (0.016; 0.031)	p=0.59, p*=0.43, p**=0.003	- 1150 (508; 2156)	- 252 (205; 293)	- 0.02 (0.014; 0.028)	- 1367 (662; 1978)	- 279 (228; 329)	- 0.021 (0.016; 0.026)	p=0.93, p*=0.07, p**=0.56	Increase in AF	Non-specific
Tyrosine	- 0 (0; 0)	- 38 (36; 43)	↓ 0.25 (0.21; 0.32)	- 0 (0; 0)	- 37 (33; 46)	↑ 0.33 (0.23; 0.47)	p=0.81, p*=0.82, p**=0.04	- 0 (0; 0)	- 38 (34; 44)	- 0.28 (0.21; 0.39)	- 0 (0; 0)	- 38 (32; 46)	- 0.3 (0.22; 0.45)	p=0.28, p*=0.70, p**=0.79	Increase in AF	Non-specific
Valine	- 0 (0; 0)	- 197 (177; 218)	↓ 0.26 (0.21; 0.31)	- 0 (0; 0)	- 210 (194; 234)	↑ 0.32 (0.25; 0.68)	p=0.34, p*=0.09, p**=0.007	- 0 (0; 0)	- 202 (189; 228)	- 0.29 (0.23; 0.49)	- 0 (0; 0)	- 203 (189; 224)	- 0.3 (0.23; 0.47)	p=0.63, p*=0.84, p**=0.99	Increase in AF	Non-specific
